# Analysis of T cell responses to chimpanzee adenovirus vectors encoding HIV gag–pol–nef antigen

**DOI:** 10.1016/j.vaccine.2015.10.111

**Published:** 2015-12-16

**Authors:** S. Herath, A. Le Heron, S. Colloca, P. Bergin, S. Patterson, J. Weber, R. Tatoud, G. Dickson

**Affiliations:** a School of Biological Sciences, Royal Holloway, University of London, Egham TW20 0EX, Surrey, UK; b ReiThera Srl, Viale Citta d’Europa 679, 00144 Rome, Italy; c Department of Immunology, Imperial College London, London, UK

**Keywords:** HIV, Vaccine, Adenovirus, Mouse, Vectors, Immunogenicity

## Abstract

Adenoviruses have been shown to be both immunogenic and efficient at presenting HIV proteins but recent trials have suggested that they may play a role in increasing the risk of HIV acquisition. This risk may be associated with the presence of pre-existing immunity to the viral vectors. Chimpanzee adenoviruses (chAd) have low seroprevalence in human populations and so reduce this risk. ChAd3 and chAd63 were used to deliver an HIV *gag*, *pol* and *nef* transgene. ELISpot analysis of T cell responses in mice showed that both chAd vectors were able to induce an immune response to Gag and Pol peptides but that only the chAd3 vector induced responses to Nef peptides. Although the route of injection did not influence the magnitude of immune responses to either chAd vector, the dose of vector did. Taken together these results demonstrate that chimpanzee adenoviruses are suitable vector candidates for the delivery of HIV proteins and could be used for an HIV vaccine and furthermore the chAd3 vector produces a broader response to the HIV transgene.

## 1 Introduction

The search for an effective, prophylactic HIV vaccine has been complicated by evidence that pre-existing immunity to some of the most commonly used human recombinant adenovirus (rAd)-based viral vectors have suppressed the effectiveness of vaccination and also may have enhanced the risk of HIV infection in some clinical trial populations [1], [2], [3]. The finding that the adenovirus subtype 5 used in the Merck STEP trial was associated with a higher incidence of HIV infection in uncircumcised males, who were seropositive for Ad5 has been linked by some investigators to the recruitment of activated CD4 T cells to the site of infection.

Many other viral and non-viral vectors have been tried and tested but have failed to provoke an efficient immune response (reviewed in [4]). Alternative human rAd HIV vaccines, based on strains less prevalent in humans have been shown to be immunogenic (reviewed in [5]). In addition, reports looking at adenoviruses from non-human species have shown that chimpanzee adenoviruses (chAds) may offer promise as potential HIV vaccines being immunogenic in mice and non-human primates [6], [7], [8].

ChAd type 3 (chAd3) and chAd type 63 (chAd63) have low seroprevalence within the populations from Africa, the Americas and India and when encoding HIV or SIV gag as a transgene were as effective as Ad5 at eliciting a T cell responses and compared favourably to alternative human rAds [9]. In addition, chAd3 encoding Gag/Pol from SIV strain mac239 induced comparable CD8 T cell immunity to rAd5 in mice and a chAd63 vectored vaccine with an antigen based on conserved HIV-1 subprotein regions has been tested and shown to be safe and immunogenic in humans [10], [11]. ChAd3 and 63 vectors have also been tested, with promising results, as vaccines for Hepatitis C, Malaria, Respiratory Syncytial Virus and Ebola [12], [13], [14], [15].

In addition to establishing an appropriate vector, suitable vaccine antigen candidates need to be identified. The spread of the HIV pandemic is largely due the virus's ability to evade immune surveillance by constantly mutating. It has been shown previously that naturally induced T cell responses to the HIV protein Gag, decreased viral load [16] and that a breadth of responses to many HIV proteins offered better protection, especially to HIV protein domains that are highly conserved and impose a survival weakness if mutated [17]. Of those proteins, Gag, Pol and Nef have more highly conserved domains and responses to these proteins have shown promise as protective vaccine candidates [16], [18], [19], [20], [21], [22].

In this study we have engineered two HIV vaccine vector candidates chAd3 and chAd63 to carry a mosaic polyprotein antigen combining epitopes from HIV proteins Gag, Pol and Nef (GPN). We have studied the immune responses to the vaccines across the gag, pol and nef elements and compared the chAd3 and chAd63 vectored vaccines as potential HIV vaccine candidates.

## 2 Materials and methods

### 2.1 Recombinant ChAd production

ChAd vector systems were provided by Okairos (Italy) and have been previously described [23], [24]. *GPN* transgene [25] was sub-cloned by homologous recombination from Okairos pShuttle (pSh) intermediate according to manufacturer's instructions.

Viruses were generated in Procell 92 cells and purified by 2 rounds of CsCl gradient ultracentrifugation and dialysed into Okairos-recommended formulation buffers. Viruses were filter sterilised, stored at −80 °C; aliquots were used once only.

Viruses were titred for infectious units (IU)/mL by infection of 293A cells followed by anti-Hexon immunoassay QuickTiter™ Adenovirus Titer Immunoassay kit (Cell Biolabs). Viruses were additionally titred for virus particles (VP)/mL by treatment with 0.1% final SDS and OD260 measurement. VP/mL are calculated as 1.1 × 10e12 VP/mL per OD260 unit [26].

### 2.2 Gene-specific PCR

Viral genome plasmid or purified viruses were treated with final 0.1% SDS at 56 °C for 10 min before transgene specific PCR. For full-length *GPN* mosaic primers were ATGGCCGCCAGAGCCTC and TCATCACTTGGCCCGGTG; *env* gene detection primers were GCCACCATGGACCGGGC and TCATCAGCTGTCCAGAGCC. For nested PCR primers for *GPN* were GGAAATCTGCGGCAAGAAGG and CTTCTTCCTCTTCCTGGGCTTC and for *env* GCCACCATGGACCGGGC and CTGCTGCTGTTGCTCTTGGT. Virus subtype matched plasmid or viral vectors encoding the *env* gene were used as negative controls. In both plasmid and viral PCRs Shuttle plasmids (pSh*GPN* and pSh*env*) known to contain the full-length *GPN* and *env* genes, respectively were used in PCR as positive, molecular weight controls.

### 2.3 Protein expression from cells infected with viral vectors

A549 cells were infected with a multiplicity of infection (MOI) of 50 IU/cell of purified viruses or transiently transfected with pSh*GPN* using Lipofectamine 2000 (Life Technologies). On d2 post-infection cells were treated with Brefeldin A (Bref A) for 4 h followed by intracellular staining using BD Cytofix/Cytoperm kit (BD Biosciences) and anti-gag PE-conjugated antibody (KC57-RD1, Beckman Coulter).

Infected cell lysates, were analysed by Western blot using Rabbit anti-HIV1 (MN) p24 anti-gag antibody (NIBSC) and secondary goat anti-rabbit IRDye^®^680RD antibody (LI-COR). Membranes were analysed using the LI-COR Odyssey^®^ scanner.

### 2.4 Mice

C57BL/6 mice were purchased from Harlan (UK); 6–8 week old females were used. All *in vivo* procedures were performed in accordance with Royal Holloway and Home Office regulations for animal experimentation.

### 2.5 Spleen cell isolation

Splenocyte suspensions were obtained using a glass homogeniser (Fisher) with RPMI containing 10% FBS, 100 IU/ml penicillin and 0.1 mg/ml streptomycin (Gibco). Cells were treated with red cell lysis buffer as directed (Sigma-Aldrich). Live cells were counted using Trypan blue exclusion.

### 2.6 Peptides

Individual HIV-ZM96 gag overlapping peptides (15-mers overlapping by 10 amino acids) were provided by NIBSC, UK and dissolved in water. Reconstituted individual overlapping peptides for HIV-ZM96 Pol 5′, Nef and Pol 3′ were provided by the International Aids Vaccine Initiative (IAVI). The peptides were pooled into peptide pool matrices such that each peptide was present in 2 pools and were used at a concentration of 1 μg/mL. Individual peptides were used at the same concentration.

### 2.7 IFNγ ELISpot

HIV specific IFN-γ T cell responses were determined by restimulation of splenocytes with peptides and quantified by anti-mouse IFN-γ ELISpot (Mabtech AB, Sweden). Spots were visualised using ABS peroxidase-avidin-biotin complexes (Vector Labs, UK) and developed by addition of AEC (Sigma Aldrich) substrate solution. Plates were dried overnight and read using an AID ELISpot reader (AutoImmun Diagnostika, Germany). Lipopolysaccharide (LPS, 1 mg/mL) and water stimulated cells served as positive and negative controls, respectively.

## 3 Results

### 3.1 Characterisation of ChAd-*GPN* plasmid and viral genomes and GPN protein expression

**Fig. 1:**
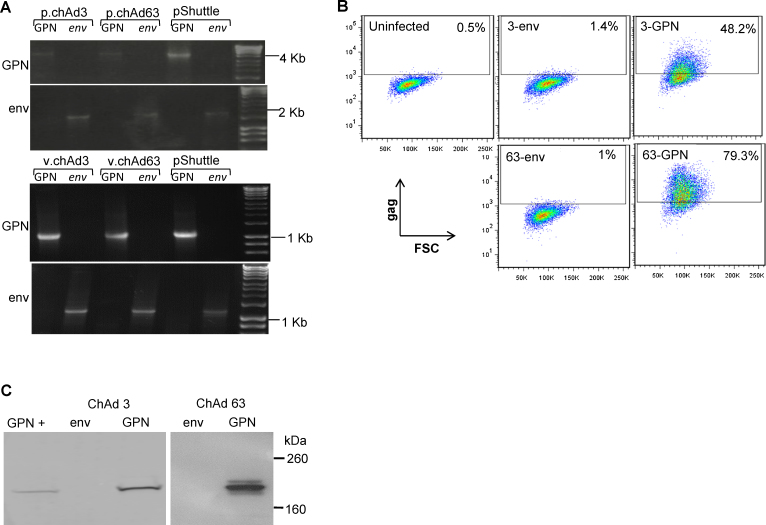
*Recombination of GPN transgene to chimp adenoviral vector plasmids and viral genomes and protein expression.* (A) Top panel: PCR analysis for full-length *GPN* gene (top gel) or negative control *env* gene (bottom gel) on chAd plasmids (p.chAd) 3-*GPN* and 63-*GPN* and subtype-matched negative control plasmids 3-*env* and 63-*env*. Bottom panel: Nested PCR analysis for full-length *GPN* gene (top gel) or negative control *env* gene (bottom gel) on purified chimp adenovirus (v.chAd) 3- and 63-*GPN* and subtype matched negative control purified chimp adenovirus 3-*env* and 63-*env*. Positive, molecular weight control in each PCR was plasmid pShuttle-*env* or pShuttle-*GPN*. (B) A549 cells were left uninfected or infected with an MOI of 50 (IU/cell) of chAd3-*GPN* and chAd63-*GPN* or negative control chAd3-*env* and 63-*env* purified viruses. On d2 post-infection cells gag protein expression was detected intracellular FACS. Percentages of PE positive cells for each infection are shown. (C) A549 cells were infected and on d2 post-infection cell lysates were analysed for gag expression by Western blot using an anti-gag p24 antibody.

To confirm successful recombination of the *GPN* gene into the chAd3 and 63 viral vectors, vector plasmids were subjected to gene-specific PCR using primers binding and amplifying the full-length *GPN* gene (Fig. 1A). Both chAd3 and 63-*GPN* plasmid genomes (Fig. 1A, top panel) and purified viruses (Fig. 1A, bottom panel) were shown to produce bands consistent with the expected 4245 bp molecular weight of the full-length *GPN* mosaic gene. No PCR amplification was seen with subtype matched, negative control chAd3 and 63-*env* viral plasmids, or viruses though bands consistent with the predicted molecular weight of the *env* gene (1968 bp) were produced by *env* specific PCR. Vector genomes were verified for integrity using restriction mapping and transgenes were sequenced (data not shown).

To ensure infectivity and GPN protein expression, A549 cells were infected with 50 IU/cell of chAd3-*GPN* or chAd63-*GPN* or as negative controls with chAd3-*env* or chad63-*env* purified viruses. Cells infected with chAd3-*GPN* and chAd63-*GPN* produced 48.2% and 79.3% Gag positive cells, respectively (Fig. 1B, right hand plots) whereas cells infected with negative control, subtype-matched *env* bearing viruses were negative for anti-gag antibody staining (middle plots).

Fig. 1C shows protein bands at 200 kDa consistent in size with that detected in pSh*GPN* transfected cells confirming that infection with these viruses drives the expression of gag-containing GPN polyprotein. GPN protein bands were not detected in negative-control subtype-matched chAd3-*env* and 63-*env* infected cells though Env protein expression was detected (data not shown). The combined FACS and western analyses show that the chAd3*-GPN* and 63-*GPN* viruses can infect cells *in vitro* and result in expression of the GPN polyprotein.

### 3.2 Response, dose and route of injection

**Fig. 2:**
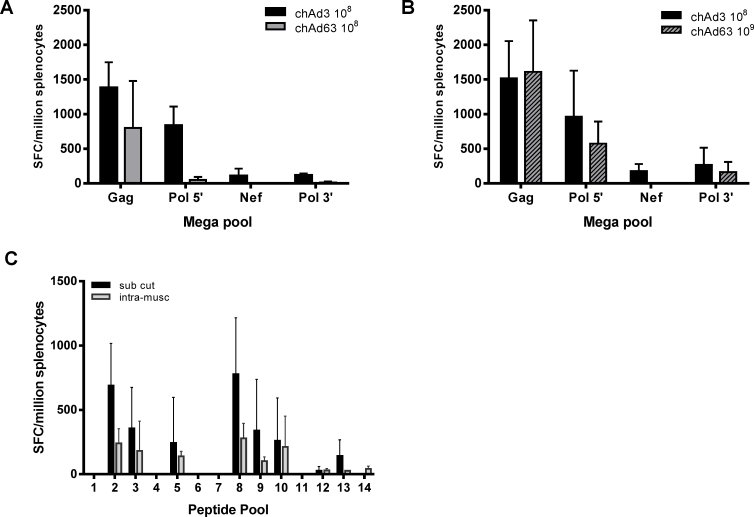
*chAd3-GPN and 63-GPN induce IFNγ responses against mega pools that are dose dependent but route independent.* Mice were vaccinated with (A) 1 × 10^8^ IU of either chAd3-*GPN* (black bar) or chAd63-*GPN* (grey bar) or with (B) 1 × 10^8^ IU chAd3-*GPN* (black bar) or 1 × 10^9^ IU chAd63-*GPN* (grey hatched bar). Splenocytes were harvested and stimulated with the indicated mega pools. (C) Mice were vaccinated with 1 × 10^8^ IU of chAd3-*GPN* either subcutaneously (black bar) or intra-muscularly (Grey bar) and splenocytes were harvested and stimulated with Gag peptide pools. IFNγ production was determined by ELISpot and graphs A and B represent 9 mice ± SD from 3 separate experiments, and graph C represents 6 mice ± SD from 2 separate experiments.

To determine whether there were immune responses to the HIV transgene, splenocytes were stimulated with mega pools, containing all the peptides, for Gag, Pol 5′, Nef and Pol 3′. Both vectors elicited responses to all mega pools except Nef, where only responses from chAd3 vaccinated animals were observed. Responses to both Pol mega pools were also higher in chAd3 injected animals (Fig. 2A). As many authors comparing vectors for vaccination inject a VP dose to determine immune responses, while we used infectious units (IU), we determined if the VP dose was similar for both vectors in our model. We analysed the VP:IU ratio in our vectors and found a tenfold difference between the two vectors (chAd3 VP:IU is 156 and chAd63 VP:IU is 14). To compensate for this, we repeated the experiment using mice vaccinated with either 1 × 10^8^ IU of chAd3-*GPN* or 1 × 10^9^ IU chAd63-*GPN*. Responses to the mega pools were more comparable at the higher dose of chAd63 though there was still no response to the Nef mega pool in chAd63 vaccinated animals (Fig. 2B). Subsequently all experiments were carried out using 1 × 10^8^ IU of chAd3-*GPN* and 1 × 10^9^ IU chAd63-*GPN*.

Previous work has shown that the route of injection can influence the immune response, due to the presence or absence of different antigen presenting cells at the site of injection. To determine whether the route of injection played a role in immune responses with chAd vectors, we injected chAd3 either subcutaneously or intra-muscularly and compared responses to gag peptide pools. Comparable responses were seen between the vaccinations suggesting that the route of injection did not significantly influence the outcome of the immune response (Fig. 2C). Consequently all vaccinations were carried out subcutaneously.

### 3.3 Immunogenicity of the chAd vectors

**Fig. 3:**
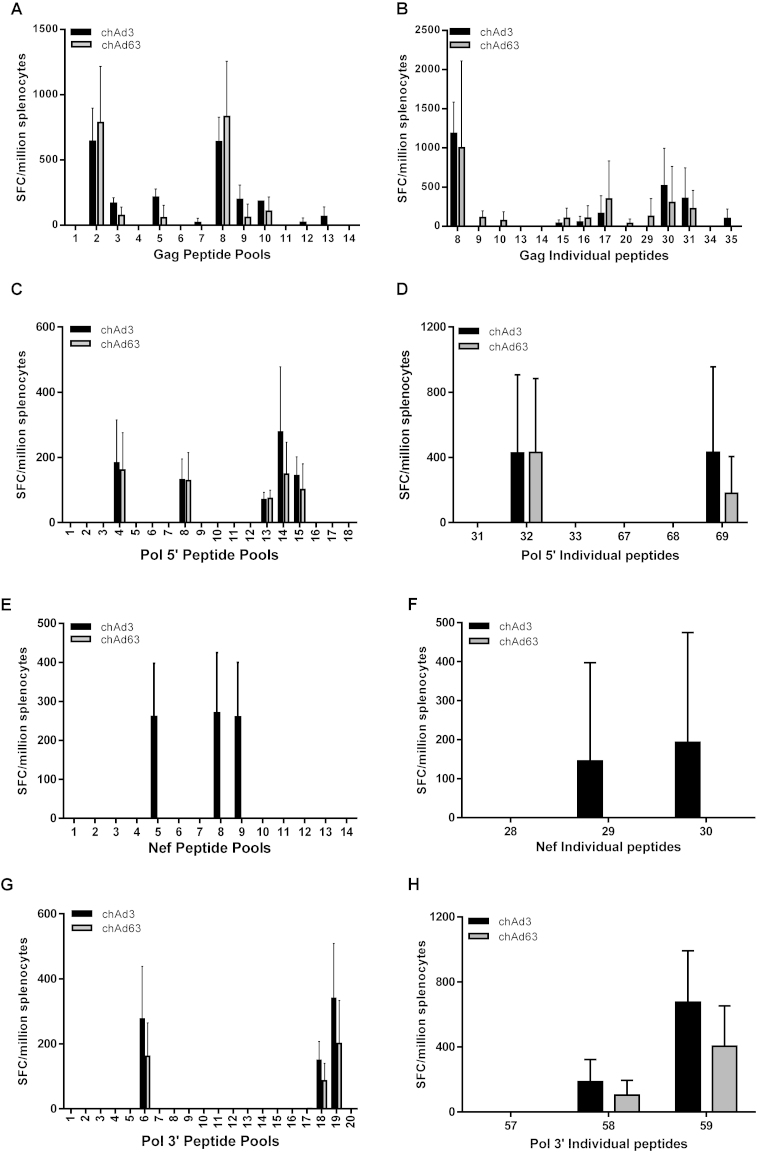
*Comparable IFNγ responses and identification of individual peptides.* Mice were vaccinated with either 1 × 10^8^ IU of chAd3-*GPN* (black bar) or 1 × 10^9^ IU of chad63-*GPN* (grey bar). Splenocytes were harvested and stimulated with the indicated peptide pools (A, C, E and G) or the individual peptides (B, D, F and H). IFNγ production was determined by ELISpot and graphs A and B represent 9 mice ± SD from 3 separate experiments and graph (C–H) represents 8 mice ± SD from 2 separate experiments.

To ascertain whether there were any specific differences in the immune response between the two vectors, mice were vaccinated with either chAd3 or chAd63 and responses to smaller peptide pools against Gag, Pol 5′, Nef and Pol 3′ were assessed. There were no differences in responses to Gag, Pol 5′ and Pol 3′ peptide pools in chAd3 and chAd63 vaccinated mice (Fig. 3A, C and G, respectively), but only chAd3 vaccinated animals responded to Nef peptide pools (Fig. 3E). To determine whether there were any differences at the epitope level, responses to individual peptides were analysed. No significant differences were observed in response to Gag, Pol 5′ and Pol 3′ individual peptides (Fig. 3B, D and H, respectively) and only chAd3 vaccinated mice responded to Nef individual peptides (Fig. 3F).

Epitope mapping of Gag, Pol and Nef was cross-checked against the individual peptide responses observed and possible epitopes were identified (Table 1). Table 1 shows that most of the possible epitopes were K^b^ restricted and 8 amino acids long. Identification of the actual epitope requires peptide synthesis along the epitope region and was beyond the scope of this work.

## 4 Discussion

Our studies aimed to determine the immunogenicity of two chAd vector vaccines encoding a transgenic antigen with potential epitopes from Gag, Pol and Nef proteins (GPN). When injected at an equal infectious unit dose, both vectors induced a response to each of the transgene products, except for Nef, and chAd3 stimulated a higher IFNγ response compared to chAd63 (Fig. 2). The magnitude of the responses in chAd63 vaccinated animals was augmented by increasing the dose of chAd63 though there was still no response to the Nef transgene product, implying that chAd63 might be less immunogenic. ChAd63 was similarly shown to be less immunogenic than ChAd3 in producing responses to SIV-gag [9].

On further analysis, it was discovered that increasing the dose of chAd63 tenfold resulted in both preparations of chAd3 and chAd63 vaccines containing a similar number of viral particles (VP) (chAd3: 1.59 × 10^10^ VP/mL and chAd63: 1.4 × 10^10^ VP/mL). During the preparation of the vectors it was noted that the chAd3 preparation resulted in an infectious unit to viral particle (IU:VP) ratio of 1:159 while the chAd63 preparation resulted in an IU:VP ratio of 1:14. Hence increasing the dose of chAd63 tenfold resulted in similar viral particle numbers and it is unclear whether defective virus particles provided an adjuvant effect. Defective adenovirus, in particular Hexon protein, has been previously shown to be a potent adjuvant [27].

The presence of IFNγ secreting cells to key peptide pools and individual peptides to each transgene product were analysed and we found that both vectors responded to the same peptides except for Nef peptides, where, interestingly, only cells from chAd3 vaccinated animals responded. There is currently no literature on the expression of the *nef* transgene in chAd vectors and we are the first, to the best of our knowledge, to identify a difference in immune responses to the Nef transgene product between two chAd vectors. Both vectors’ transgenes have been fully sequenced and have been shown to produce proteins consistent in size with the full-length GPN transgene (Fig. 1C). However, we have not statistically quantified the level and duration of expression of GPN from each vector and expression of antigen transcripts was seen to vary between chAd3 and 63 vectors expressing SIV-gag [10].

Computational analysis of the epitopes expressed by the C57BL/6 mouse strain for Gag, Pol 5′, Nef and Pol 3′ indicated that some peptides contained more than 1 epitope (Table 1). Analysis of our findings suggest that vaccination with chAd vectors results in the presentation of a dominant peptide in the Gag and Nef transgene product (peptide 8 for Gag and peptide 59 for Nef) due to the higher responses observed compared to the other peptides within the transgene. The limitations of our experimental design do not make it possible to determine which epitopes are being expressed as this would require designing pentamers against each potential epitope.

Taken together our results showed that both chAd3 and chAd63 induced responses against a maximum of 6 epitopes within HIV Gag and 5 epitopes within Pol with 3 further epitopes within Nef for chAd3. It is well recognised that HIV circulate with large genetic variability within strains and the requirement of a suitable prophylactic vaccine would be to induce a broad response to many epitopes implying that chAd3 would be a better vaccine vector. However, it must be noted that our experiments did not investigate the polyfunctionality of the responding cells nor did we determine the magnitude of innate immune responses. Quinn *et al.* [9] show that both chAd3 and chAd63 induce a low frequency of IFNγ-only producing T cells but a high percentage of polyfunctional T cells to a dominant HIV gag peptide. Furthermore, variation of type I IFN responses between different rAd vectors was reported and correlated to immunogenicity [10]. Consequently both chAd3 and chAd63 could elicit immune responses not measured in this study. The fact that both vectors recognise similar epitopes diminishes the possibility of both vectors being used in a single vaccine to broaden the response to the transgene product.

In addition to designing a vaccine, the route of injection is also an important factor in assessing efficacy [28], [29], [30], [31], [32]. To this end we determined whether the chAd vectors induced differing responses when injected subcutaneously or intramuscularly. In line with previous work, there was no significant difference in the magnitude or breadth of IFNγ responses observed with either route of injection (Fig. 2) [30], [33]. Professional antigen presenting cells are abundant in the subcutaneous layer of the skin but are less numerous in muscle tissue [34]. However the observation that both vectors induce a similar response despite the route of injection suggest that both vectors are recognised in muscle tissue. Intramuscular injections are preferred as a vaccination route due to the adequate blood supply to the muscle, which aids in the dispersion of the product. The result that both vectors induced an immune response in muscle tissue bodes well for chAd vectors being used in intramuscular injections.

Like other adenoviruses, both chimpanzee adenovirus vectors induced an immune response to the transgene products Gag and Pol, with hierarchal epitope dominance seen in the Gag product. Both vectors responded to similar epitopes for Gag and Pol elements and the route of injection did not alter the outcome of immune responses. Interestingly, only chAd3 vaccination induced responses to the Nef transgene product suggesting that chAd3 may be superior as a vaccine vector candidate. Taken together, our results suggest that chimpanzee adenoviruses with their low global seroprevalance demonstrable immunogenicity in various species and safety in human trials to date could be good vaccine candidates as viral vectors for HIV vaccines.

## Figures and Tables

**Table 1:** *Identification of possible epitopes.* Computational analysis (http://www.mpiib-berlin.mpg.de/MAPPP/) of possible epitopes by C57BL/6 mice against the HIV transgene were cross-checked against the peptide sequence of individual peptides that were positive for IFN-γ production.

Transgene	Peptide no.	Peptide sequence	Possible epitope	Restriction
Gag	8	GTEELRSLYNTVATLYCVHE	GTEELRSL/LRSLYNTVA	H2K^b^/H2K^b^
17	EKAFSPEVIPMFTALSEGAT	FSPEVIPMF/VIPMFTAL	H2K^b^/H2K^b^
30	EPFRDYVDRFFKTLRAEQAT	VDRFFKTLR	H2K^b^
31	FKTLRAEQATQEVKNWMTDT	QEVKNWMTD	H2K^b^
Pol 5′	32	GTVLVGPTPVNIIGR	VGPTPVNII	H2D^b^
69	ESFRKYTAFTIPSTN	RKYTAFTI	H2K^b^
Nef	29	GKWSKSSIVGWPAVR	KWSKSSIV/SIVGWPAV	H2K^b^/H2K^b^
30	KSSIVGWPAVRERIR	SIVGWPAV	H2K^b^
Pol 3′	58	IVGAETFYVDGAANR	GAETFYVD/AETFYVDG	H2K^b^/H2K^b^
59	ETFYVDGAANRETKL	AANRETKL	H2K^b^
